# Conserved Architecture, Divergent Function: Tomato Lunapark Proteins Remodel the Endoplasmic Reticulum Without Enhancing Pathogen Resistance

**DOI:** 10.3390/plants15213447

**Published:** 2026-11-05

**Authors:** Dan Jackson, Carmen Mata, Nadine Field, Lorena Pizarro, Adi Avni, Stephen C. Whisson, Keith Miller, Stanley W. Botchway, Charlotte Pain, Verena Kriechbaumer

**Affiliations:** 1 Endomembrane Structure and Function Research Group, School of Biological and Medical Sciences, Oxford Brookes University, Gipsy Lane, Oxford OX3 0BP, UK; 19066745@brookes.ac.uk (D.J.); 19276303@brookes.ac.uk (C.M.); 19180311@brookes.ac.uk (N.F.); p0089599@brookes.ac.uk (C.P.); 2 Oxford Brookes Centre for Bioimaging, Oxford Brookes University, Gipsy Lane, Oxford OX3 0BP, UK; 3 School of Plant Sciences and Food Security, Tel-Aviv University, Ramat-Aviv, Tel Aviv 69978, Israel; lorena.pizarro@uoh.cl (L.P.); lpavni@tauex.tau.ac.il (A.A.); 4 Instituto de Ciencias Agroalimentarias, Animales y Ambientales, Universidad de O’Higgins, San Fernando 3070000, Chile; 5 Cell and Molecular Sciences Department, The James Hutton Institute, Invergowrie, Dundee DD2 5DA, UK; steve.whisson@hutton.ac.uk; 6 School of Biosciences and Chemistry, Sheffield Hallam University, Sheffield S1 1WB, UK; k.miller@shu.ac.uk; 7 Central Laser Facility, Research Complex at Harwell, Rutherford Appleton Laboratory, Science and Technology Facilities Council, Didcot OX11 0QX, UK; stan.botchway@stfc.ac.uk

**Keywords:** AnalyzER, endoplasmic reticulum, FRET-FLIM, Lunapark, *P. infestans*, secretion, tomato

## Abstract

The endoplasmic reticulum (ER) is a dynamic membrane network that plays essential roles in protein secretion, lipid biosynthesis and cellular signalling. Its architecture is maintained by conserved ER-shaping proteins, including the recently identified Lunapark (LNP) family, which have primarily been characterised in *Arabidopsis thaliana*. Whether these mechanisms are conserved in crop species remains unknown. Here, we identify and characterise two tomato (*Solanum lycopersicum*) LNP proteins, *Sly*LNP1 and *Sly*LNP2, and investigate their roles in ER organisation and function. Both proteins localised to the ER but displayed distinct distribution patterns, with *Sly*LNP1 preferentially associated with cisternae and *Sly*LNP2 localising to both cisternae and tubules. Overexpression of either protein promoted cisternal expansion and altered ER network organisation. FRET-FLIM analyses demonstrated that both *Sly*LNP proteins interact with themselves, each other, the ER fusogen RHD3 and the membrane-curvature protein RTN1, indicating conservation of the ER-shaping machinery. Furthermore, *Sly*LNP overexpression reduced ER-to-Golgi trafficking, linking ER remodelling with secretory pathway function. In contrast to their Arabidopsis counterparts, however, expression of tomato LNP proteins in *Nicotiana benthamiana* did not reduce infection by *Phytophthora infestans*. Together, our findings demonstrate that the molecular mechanisms governing ER architecture are highly conserved in tomato while revealing that the physiological consequences of Lunapark-mediated ER remodelling have diverged between plant species.

## 1. Introduction

### 1.1. The Endoplasmic Reticulum Is a Dynamic Regulator of Plant Cell Function

The endoplasmic reticulum (ER) is one of the largest and most dynamic membrane systems in eukaryotic cells, forming a continuous network of interconnected tubules and cisternae that extends throughout the cytoplasm and is continuous with the nuclear envelope [1,2]. As the entry point to the secretory pathway, the ER is responsible for the synthesis, folding and quality control of secretory and membrane proteins, lipid biosynthesis and calcium storage, making it essential for plant growth, development and responses to environmental stimuli [3,4]. Protein export from the ER occurs through specialised ER exit sites (ERES), which form dynamic secretory units with closely associated Golgi bodies and enable efficient trafficking of newly synthesised proteins to downstream compartments [5,6,7]. Consequently, ER morphology is intimately linked to ER function, and perturbations in ER organisation can have profound effects on intracellular trafficking and cellular homeostasis.

### 1.2. Conserved ER-Shaping Proteins Maintain the Balance Between Tubules and Cisternae

The architecture of the ER is maintained through the coordinated activities of several conserved membrane-shaping protein families. Reticulons (RTNs) stabilise highly curved membranes and promote tubule formation [8,9,10,11,12,13], whereas ROOT HAIR DEFECTIVE 3 (RHD3), the plant orthologue of the metazoan atlastins and yeast Sey1p, mediates homotypic membrane fusion required for formation and maintenance of the interconnected ER network [14,15,16,17,18,19,20,21,22,23]. Together, these proteins establish the dynamic equilibrium between tubular and cisternal ER domains that underpins ER organisation and function. A third family of ER-shaping proteins, the Lunapark (LNP) proteins, has emerged as an important regulator of ER morphology. LNP proteins are characterised by two N-terminal transmembrane domains (TMDs), a conserved C-terminal zinc-finger domain and a characteristic LNPARK motif or related sequence [24,25,26,27,28,29,30,31,32]. In yeast and mammalian cells, LNP proteins predominantly localise to ER three-way junctions, where they interact with reticulons and the reticulon-like protein Yop1p, which promote membrane curvature and tubule formation [25,33]. In *Saccharomyces cerevisiae*, Lnp1p acts antagonistically to the dynamin-like GTPase Sey1p, which mediates ER membrane fusion, suggesting that LNP proteins help regulate the balance between junction formation and remodelling of the ER network [25]. In mammalian cells, LNP1 similarly contributes to the stabilisation of nascent ER three-way junctions [26]. These findings support a role for LNP proteins in coordinating membrane curvature, fusion and junction stability. However, the mechanisms and functional outcomes of LNP activity may differ between organisms, as emerging evidence from plants indicates that plant LNP proteins also play important roles in ER cisternal organisation [28,29].

### 1.3. Plant LNP Proteins Promote ER Cisternal Organisation

Arabidopsis contains two LNP homologues, *At*LNP1 and *At*LNP2, that differ markedly from their yeast and mammalian counterparts. Rather than localising predominantly to three-way junctions, *At*LNP1 is enriched within ER cisternae, while *At*LNP2 labels both cisternae and tubular ER [28]. Loss of LNP function reduces cisternal abundance, whereas overexpression promotes extensive cisternal expansion, suggesting that plant LNP proteins contribute to ER cisternal organisation [28]. These findings initially suggested that plant LNP proteins primarily promote the formation or expansion of ER sheets, distinguishing their apparent role from the regulation of ER three-way junctions described in yeast and mammalian cells. Furthermore, AtLNP proteins physically interact with RHD3 and RTN1, suggesting that they function together with other ER-shaping proteins to coordinate membrane fusion and membrane curvature during ER remodelling [28,31]. More recent ultrastructural analyses have substantially refined this model. Using correlative light and electron microscopy combined with electron tomography, these studies demonstrated that LNP-induced cisternae are not simply flattened membrane sheets but comprise densely interconnected tubular matrices surrounded by peripheral membrane sheets [29]. Importantly, these structural changes were accompanied by reduced ER-to-Golgi transport, establishing a functional relationship between LNP-mediated ER remodelling and secretory pathway efficiency [29]. Together, these findings suggest that plant LNP proteins regulate a complex ER architecture in which tubular and sheet-like membrane domains are organised together, with consequences for secretory pathway function.

### 1.4. Conservation of LNP Function in Crop Species Remains Unknown

Despite these advances, our understanding of plant LNP function remains largely restricted to Arabidopsis. Whether the mechanisms governing ER organisation are conserved in agriculturally important crop species has not been investigated. This represents a significant knowledge gap because crop plants exhibit distinct developmental programmes, physiological demands and secretory requirements that may influence ER organisation. Understanding whether LNP proteins perform equivalent functions outside Arabidopsis is therefore essential for determining whether current models of plant ER organisation can be generalised and for assessing their potential application in crop improvement. Tomato (*Solanum lycopersicum*) is an excellent system within the Solanaceae family in which the roles of LNP proteins in ER organisation have not yet been characterised. As one of the world’s most important horticultural crops and a well-established model for Solanaceae biology, tomato provides an opportunity to examine whether conserved ER-shaping mechanisms operate within a species of considerable agricultural importance [34].

ER organisation may also influence plant immunity through its role in the synthesis, folding and trafficking of defence-related proteins. The ER supports the biogenesis of membrane-localised pattern-recognition receptors and the production of secreted defence proteins, while ER-to-Golgi trafficking is required for their delivery to appropriate cellular locations [35,36]. Consequently, changes in ER architecture could affect the efficiency of secretory processes that contribute to immune responses. As LNP proteins remodel ER morphology and influence ER-to-Golgi transport [28,29], they may indirectly affect plant–pathogen interactions. However, whether these effects on ER organisation translate into altered pathogen resistance, and whether this relationship is conserved between plant species, remains unclear.

Here, we investigate the two tomato LNP proteins, *Sly*LNP1 and *Sly*LNP2, to determine the extent to which their cellular functions are conserved with those previously described in Arabidopsis. Using quantitative live-cell imaging, we examine their subcellular localisation and effects on ER architecture, investigate their interactions with the conserved ER-shaping proteins RHD3 and RTN1 using Förster resonance energy transfer coupled with fluorescence lifetime imaging microscopy (FRET-FLIM), and assess their effects on ER-to-Golgi trafficking. We also investigate whether the expression of tomato LNP proteins influences susceptibility to the oomycete pathogen *Phytophthora infestans*. We hypothesised that tomato LNP proteins would share key ER-remodelling properties with their Arabidopsis counterparts, but that these conserved cellular functions might not necessarily result in equivalent effects on pathogen resistance.

## 2. Results

### 2.1. Tomato Lunapark Proteins Share Conserved Structural Features with LNP Homologues

**Figure 1:**
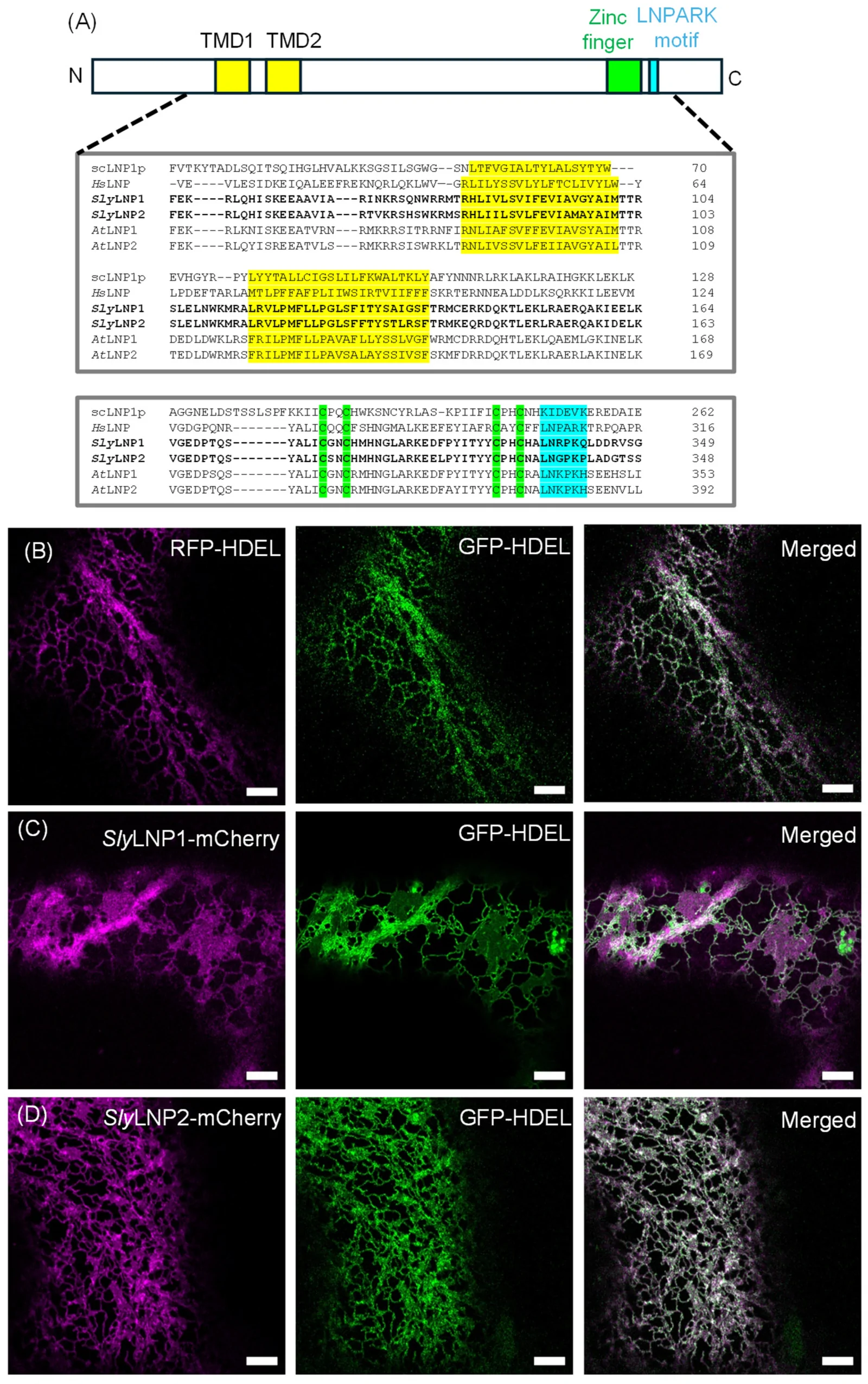
Domain architecture and subcellular localisation of tomato LNP (*Sly*LNP) proteins. (**A**) Partial amino acid alignment focusing on highlighting key structural features: the two predicted TMDs (TMD1, TMD2), the C-terminal zinc finger (four conserved cysteines, shaded green), and the LNPARK motif. In Arabidopsis, the motif is “LNKPKH”; in tomato, “LN[G/R]PK[P/Q]”; while *S. cerevisiae* lacks an obvious LNPARK sequence. The alignment shows selected regions of the sequences and does not represent the full-length proteins. The full alignment is provided in Supplementary Figure S1. (**B**) Localisation of ER markers: GFP-HDEL (green) and RFP-HDEL (magenta) co-expressed in Agrobacterium-transformed tobacco epidermal cells. (**C**) Co-localisation of *Sly*LNP1-mCherry (magenta) with GFP-HDEL (green), showing enrichment in ER cisternae. (**D**) Co-localisation of *Sly*LNP2-mCherry (magenta) with GFP-HDEL (green), showing uniform ER network distribution. Scale bars = 5 µm.

We compared the tomato LNP proteins *Sly*LNP1 and *Sly*LNP2 with characterised LNP homologues, including Arabidopsis *At*LNP1 and *At*LNP2, human LNP (*Hs*LNP) and the single-copy *Saccharomyces cerevisiae* Lunapark protein ScLNP1p (Figure 1A; Supplementary Figure S1). *Sly*LNP1 and *Sly*LNP2 are very highly conserved, with 76% sequence identity, particularly through the N-terminal ~300 aa. They are 405 aa (*Sly*LNP1) and 400 aa (*Sly*LNP2), and the sequences show almost complete conservation of the characteristic two N-terminal TMDs, followed by a highly conserved cytoplasmic region. The two TMDs are essentially identical in position and composition, with only a few conservative substitutions. The domain organisation of tomato LNP proteins closely resembles the Arabidopsis LNP family members: they possess two predicted TMDs near the N-terminus (Figure 1A; Supplementary Figure S1), as well as a C-terminal Cys_4_-type zinc finger followed by the characteristic LNPARK motif [25,28,29]. The greatest sequence divergence occurs in the LNP N-terminal extensions and, especially, the low-complexity C-terminal tails, which are rich in Ser/Ala/Gly/Pro and differ considerably in length and composition. Interestingly, the zinc finger is almost invariant, whereas the adjacent LNP motif shows species-specific variation. Although the LNPARK motif remains recognisable in the tomato proteins, it has diverged substantially at the amino acid level, LNRPKQ (*Sly*LNP1) and LNGPKP (*Sly*LNP2), while the four conserved cysteines of the zinc-finger region are maintained. Previous studies in Arabidopsis have shown that LNP proteins integrate into the ER membrane via their N-terminal TMDs and predominantly localise to the ER [25,28]. The ER can be efficiently visualised in Agrobacterium-transformed cells using fluorescent markers such as RFP (red fluorescent protein)-HDEL or GFP (green fluorescent protein)-HDEL [37] (Figure 1B).

Tomato LNP1 and LNP2 were transiently expressed in *Nicotiana tabacum* to investigate their subcellular localisation and effects on endoplasmic reticulum (ER) organisation in a plant cellular environment. As members of the Solanaceae, tomato and tobacco share aspects of their cellular organisation and secretory machinery, making tobacco a practical heterologous system for examining the behaviour of tomato proteins in plant cells. This approach enables the analysis of protein localisation and ER morphology, although it does not by itself establish that protein interactions or functions are identical in tomato. Accordingly, we used this system to assess the extent to which the subcellular behaviour and ER-related functions of tomato LNP proteins are consistent with conserved roles described in other plant species.

When we expressed *Sly*LNP1-mCherry in tobacco leaf epidermal cells, the fusion protein co-localised with the ER marker and displayed particularly strong accumulation in ER cisternae (Figure 1C). In contrast, *Sly*LNP2-mCherry was distributed more uniformly throughout the ER network (Figure 1D). These localisation patterns mirror those previously reported for *At*LNP1 and *At*LNP2, respectively [28,29]. Together, these findings show that tomato LNP proteins share selected structural features and subcellular localisation patterns with their Arabidopsis counterparts, consistent with conservation of aspects of their cellular functions.

### 2.2. Tomato LNP Proteins Remodel ER Architecture

**Figure 2:**
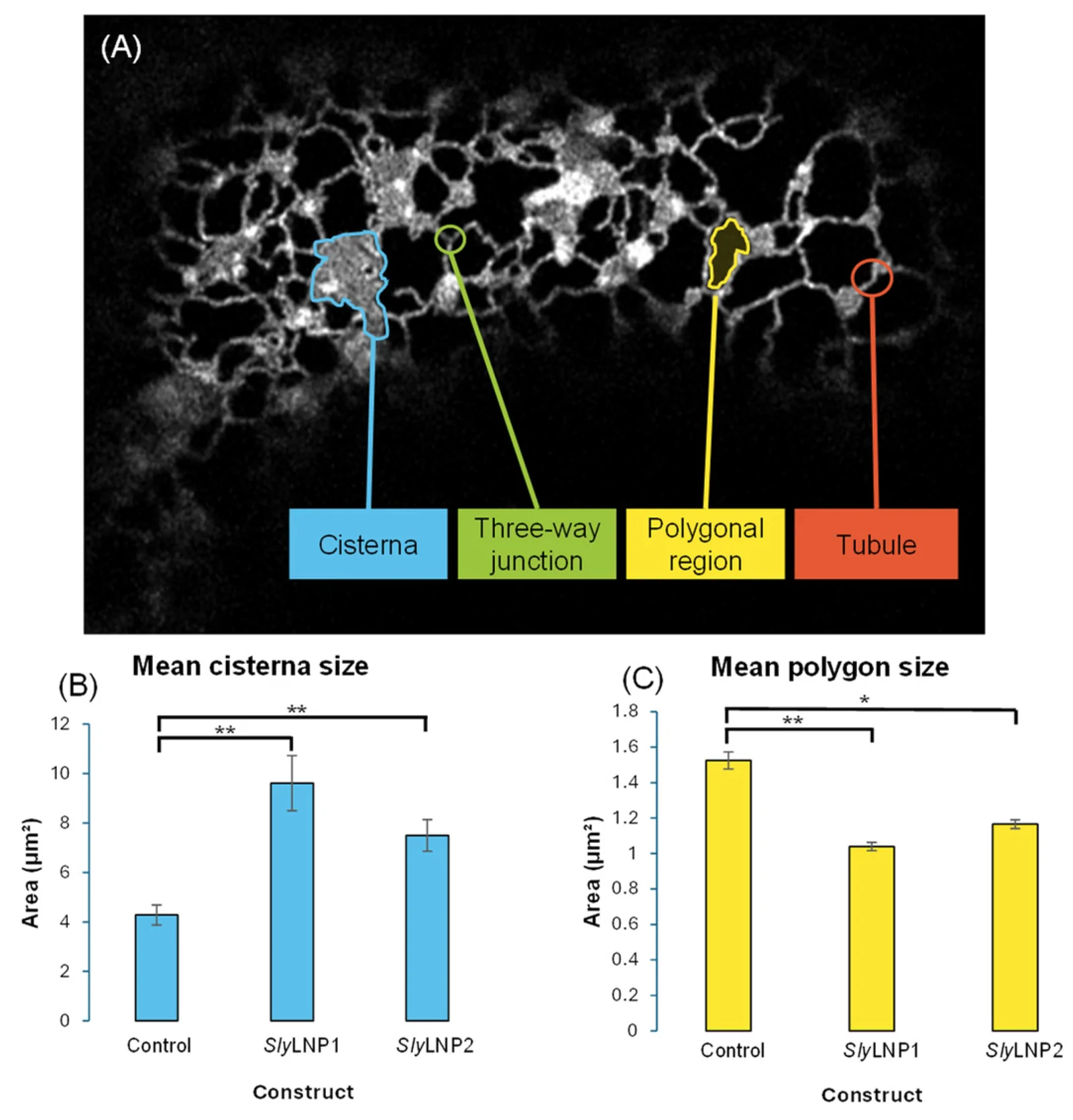
Impact of *Sly*LNP1 and *Sly*LNP2 overexpression on ER morphology. (**A**) Schematic of ER subdomains: cisternae (blue), tubules (red), three-way junctions where tubules converge (green), and polygonal cytoplasmic regions enclosed by the ER network (yellow). (**B**) Quantification of mean cisternal area under overexpression of *Sly*LNP1-mCherry and *Sly*LNP2-mCherry compared to control (RFP-HDEL). (**C**) Quantification of mean polygonal region size under *Sly*LNP1-mCherry and *Sly*LNP2-mCherry overexpression versus control (RFP-HDEL). Sample sizes: control, *n* = 17; *Sly*LNP1, *n* = 24; *Sly*LNP2, *n* = 26; data derived from three independent biological replicates. Significant differences between groups were identified using Tukey’s test, where ** indicates a *p*-value < 0.01 and * indicates a *p*-value < 0.05. Scale bars = 5 µm.

The ER network can be divided into two principal structural subdomains: cisternae, which are broad, flattened sheet-like regions, and tubules, which are narrow membrane strands forming the network (Figure 2A). Tubules may connect to cisternae, to each other via three-way junctions, or terminate as free ends, with the latter potentially contributing dynamically to ER remodelling [1,38,39,40]. The cytoplasmic regions enclosed by this network are known as polygonal regions; these negatively defined spaces provide valuable insight into the ER’s overall geometry.

To quantify how overexpression of tomato LNP proteins impacts ER architecture, we employed the AnalyzER V1.1 software package to measure nine distinct metrics of ER structure and network geometry [27,41,42]. AnalyzER is specifically designed to extract quantitative morphological and topological features from fluorescence images of the ER.

First, we performed a multivariate analysis of variance (MANOVA) to assess whether the global network metrics differed between experimental groups (Pillai’s trace, *p* = 4.76 × 10^−4^; Roy’s largest root, *p* = 6.17 × 10^−6^). Having established significant overall differences, we conducted univariate ANOVAs on each metric, correcting *p*-values for multiple comparisons using the Bonferroni method (Supplementary Table S1). For those measures showing significant effects, we carried out post hoc pairwise comparisons using Tukey’s test (Supplementary Table S2; Figure 2B,C). These analyses revealed that overexpression of *Sly*LNP1 led to a 124% increase in the mean cisternal area relative to control cells, while *Sly*LNP2 overexpression produced a 75% increase. Conversely, the average size of polygonal regions decreased by 32% with *Sly*LNP1 overexpression and by 24% with *Sly*LNP2, indicating a substantial reorganisation of the ER network under both conditions. These results indicate that both tomato LNP proteins promote ER cisternal expansion and remodel the overall ER network architecture, consistent with a conserved role in ER cisterna organisation.

### 2.3. Overexpression of SlyLNP1 and SlyLNP2 Induces Distinct Cisternae Architectures

**Figure 3:**
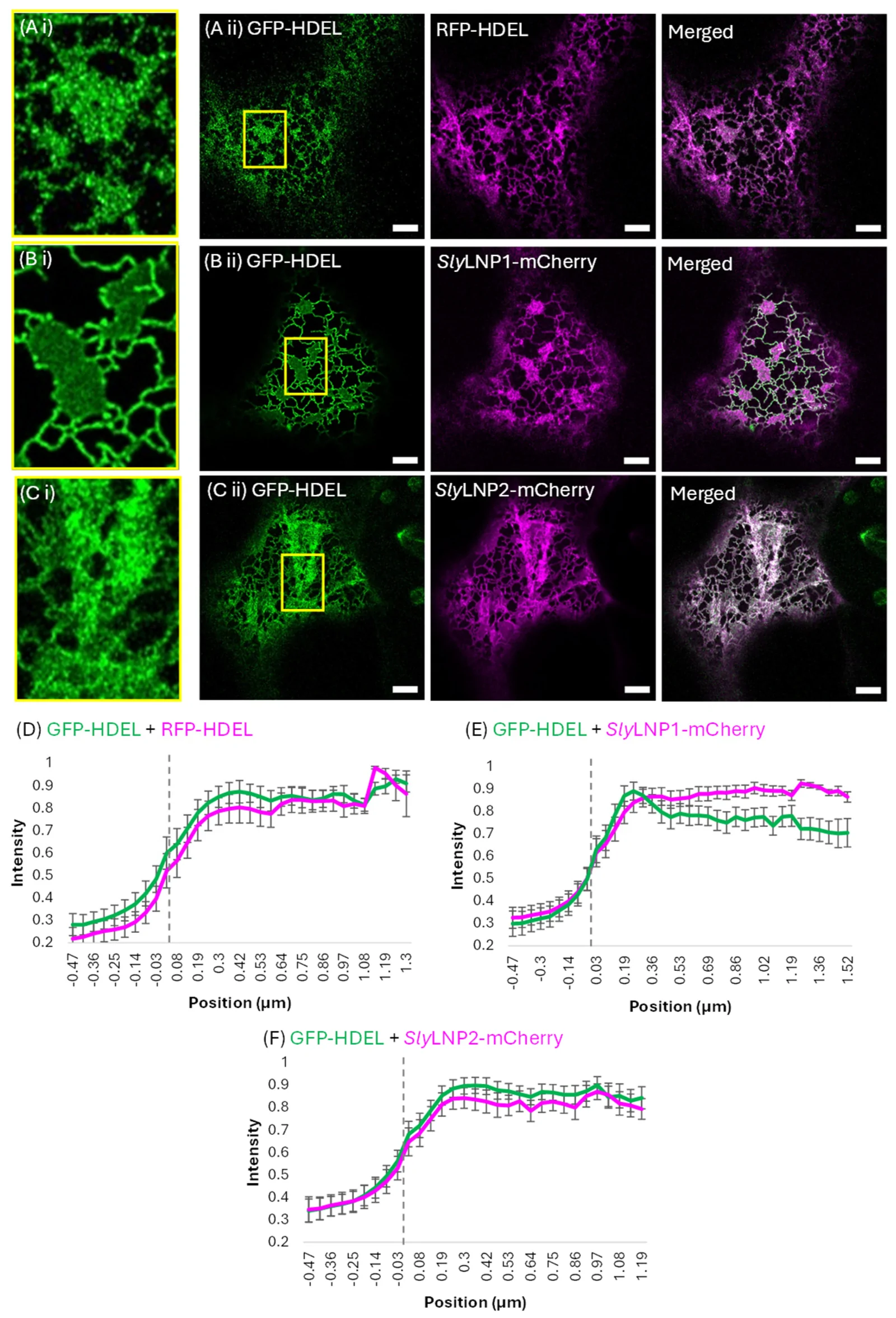
Close-up and intensity profiling of ER cisternae under different overexpression conditions. (**Ai**–**Ci**) Magnified views of a representative cisterna (yellow box) in cells expressing: (**Ai**) GFP-HDEL (green) plus RFP-HDEL (magenta) in control, (**Bi**) *Sly*LNP1-mCherry (magenta), or (**Ci**) *Sly*LNP2-mCherry (magenta). (**Aii**–**Cii**) Corresponding full-cell images showing co-expression of GFP-HDEL with (**Aii**) RFP-HDEL control, (**Bii**) *Sly*LNP1-mCherry, or (**Cii**) *Sly*LNP2-mCherry. Scale bars for (**A**–**C**) = 5 µm. (**D**–**F**) Mean fluorescence intensity profiles across the cisternal boundary (from −0.47 µm outside to the centre). The dashed outline indicates the cisternal perimeter identified by AnalyzER using “thresh1” segmentation with active-contour refinement. The lumenal marker (GFP-HDEL) is plotted in green; the magenta traces show (**D**) RFP-HDEL in control, (**E**) *Sly*LNP1-mCherry, and (**F**) *Sly*LNP2-mCherry. Data are means from *n* = 7 (control), *n* = 9 (*Sly*LNP1), and *n* = 11 (*Sly*LNP2) cells, collected from three independent biological replicates. Error bars represent ± SEM.

Overexpression of *Sly*LNP1 and *Sly*LNP2 alters ER cisternae in quantitatively similar but structurally distinct ways. While both proteins increase mean cisternal area (Figure 2), their localisation patterns differ markedly: *Sly*LNP1 preferentially labels cisternae, whereas *Sly*LNP2 is more uniformly distributed throughout the ER network. Notably, in cells overexpressing *Sly*LNP1, the luminal marker GFP-HDEL is displaced toward the edges of cisternae (Figure 3B), unlike in control cells (Figure 3A) or those overexpressing *Sly*LNP2 (Figure 3C), where GFP-HDEL fills the cisternal interior more evenly. Intensity profiling across the cisternal cross-sections reveals a pronounced peak of green fluorescence at the periphery in *Sly*LNP1-overexpressing cells (Figure 3E), a feature not observed under control conditions or with *Sly*LNP2 overexpression (Figure 3D,F). This suggests that *Sly*LNP1 overexpression reorganises the distribution of luminal contents within cisternae, a phenomenon not recapitulated by *Sly*LNP2.

### 2.4. Secretion Assays Showed SlyLNP Proteins Differentially Affect ER-to-Golgi Transport

**Figure 4:**
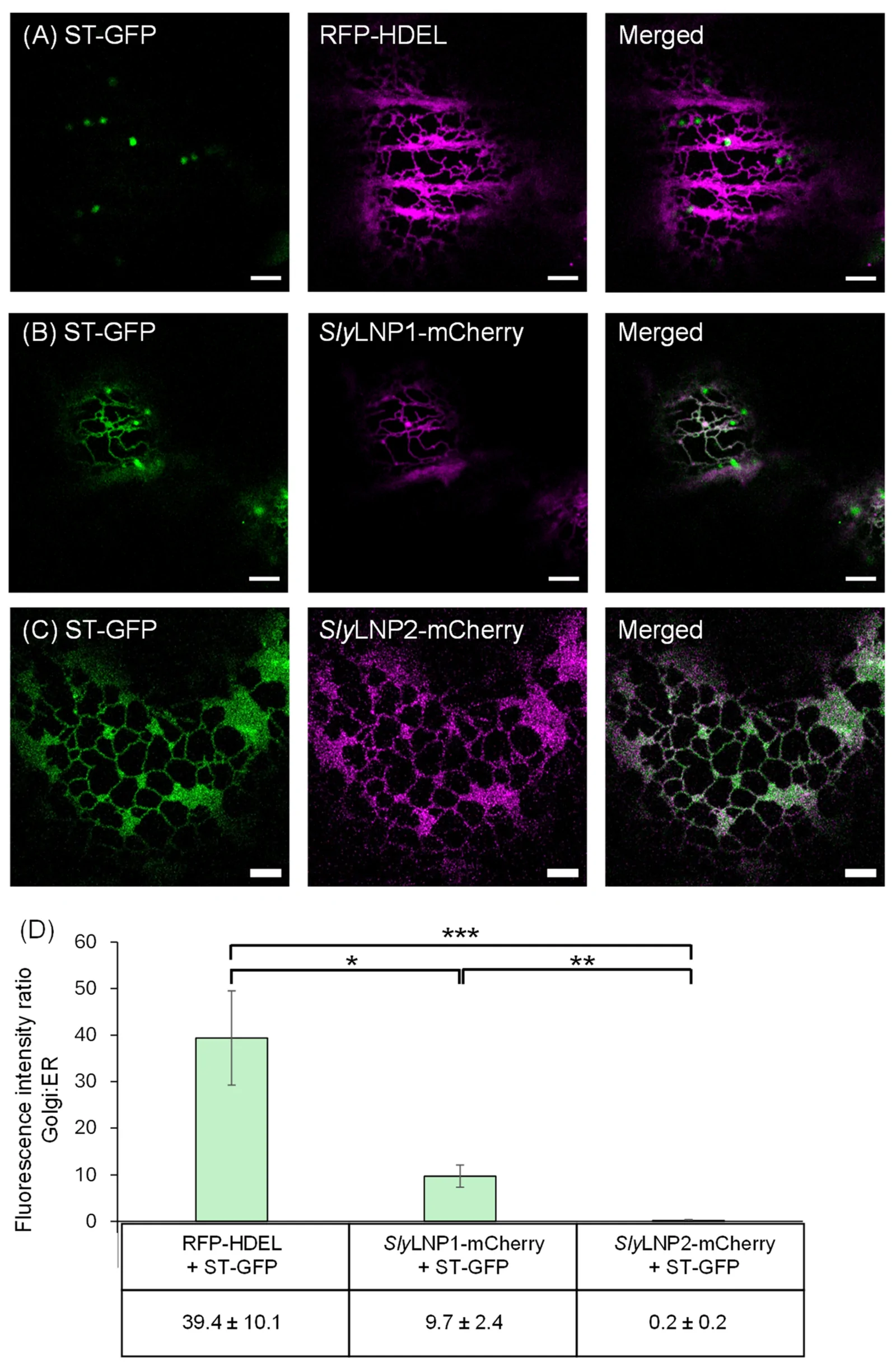
Overexpression of *Sly*LNP1 and 2 reduces transport between the ER and Golgi bodies. Representative images of overexpression of the Golgi-localised marker ST-GFP (green) alongside (**A**) an ER-localised control (RFP-HDEL, magenta), (**B**) *Sly*LNP1-mCherry (magenta), and (**C**) *Sly*LNP2-mCherry (magenta). Individual channel images are shown alongside merged channel images. Scale bars = 5 μm. (**D**) Quantification of Golgi/ER fluorescence intensity ratio as a proxy for blocked ER-to-Golgi body transport. Data were collected from *n* = 11 (control), *n* = 12 (*Sly*LNP1), and *n* = 14 (*Sly*LNP2) cells, collected from three independent biological replicates. Error bars represent ± SEM. Post hoc comparisons were performed using Dunn’s test and the Kruskal–Wallis test. *** indicates a *p*-value < 0.001, ** indicates a *p*-value < 0.01 and * indicates a *p*-value < 0.05.

To investigate whether the changes in ER morphology induced by *Sly*LNP overexpression affected secretory pathway function, ER-to-Golgi trafficking was assessed using the Golgi body marker Sialyl Transferase (ST)-GFP [43,44]. In control cells expressing RFP-HDEL, ST-GFP displayed the characteristic Golgi body localisation (Figure 4A). In contrast, co-expression of either *Sly*LNP1-mCherry or *Sly*LNP2-mCherry resulted in increased retention of ST-GFP within the ER and a corresponding reduction in Golgi body localisation (Figure 4B,C), indicating impaired ER export. Quantitative analysis confirmed that overexpression of both *Sly*LNP proteins significantly reduced ER-to-Golgi transport compared with the control (Figure 4D). Although both proteins inhibited trafficking, no significant difference was detected between *Sly*LNP1 and *Sly*LNP2, suggesting that both LNP homologues similarly influence early secretory pathway function. These observations indicate that the extensive ER remodelling induced by increased *Sly*LNP abundance has functional consequences for intracellular membrane trafficking, supporting a close relationship between ER architecture and protein export. Together with the observed increase in ER cisternal abundance, these results suggest that excessive LNP-mediated stabilisation of ER cisternae alters the organisation of the early secretory pathway and consequently reduces the efficiency of protein export from the ER.

### 2.5. FRET-FLIM Indicated Interactions Between Tomato ER-Shaping Proteins

**Figure 5:**
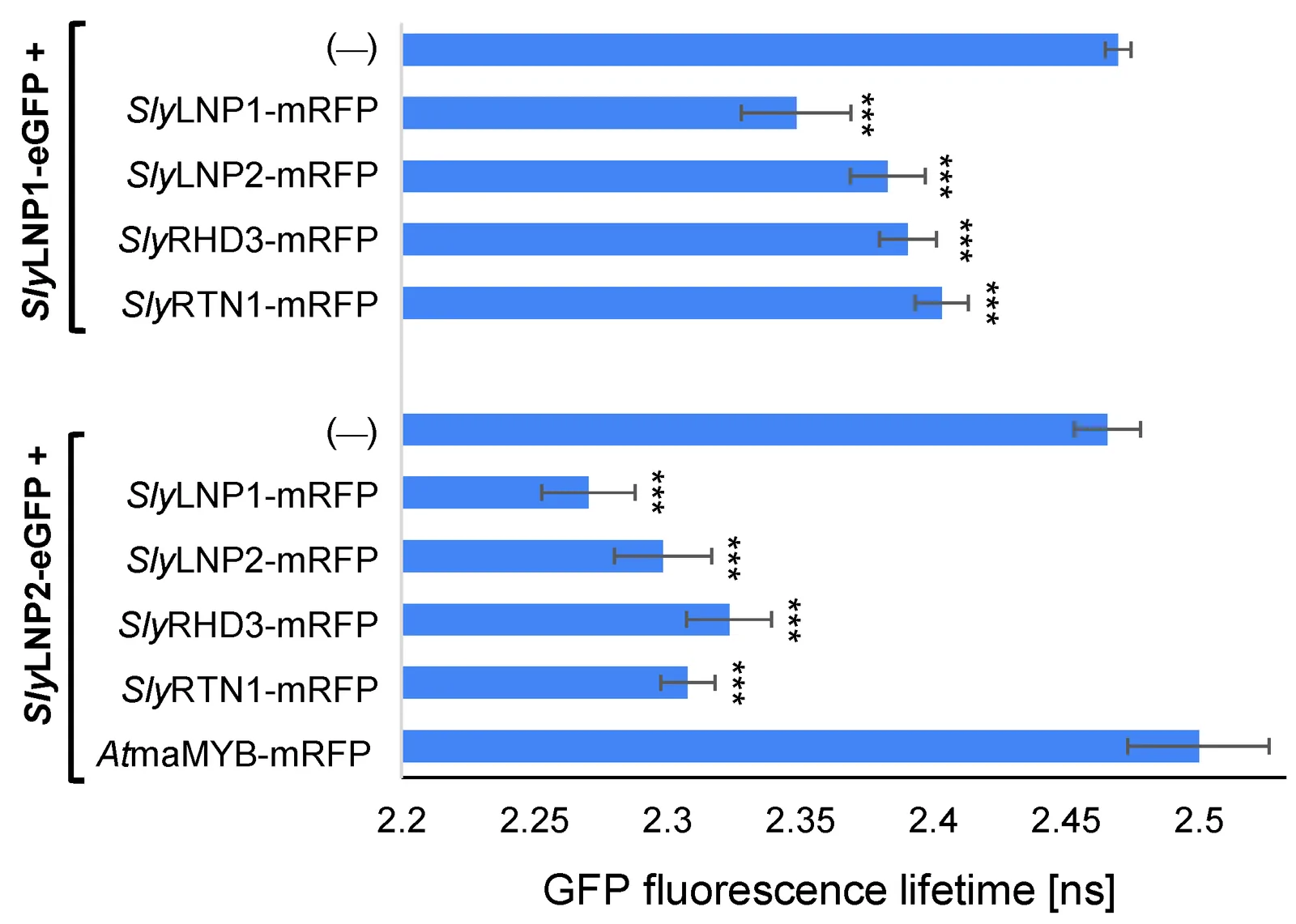
Fluorescence lifetimes in FRET-FLIM interactions between *Sly*LNP1 and 2 and other shapers. The bar graphs represent average fluorescence lifetimes [ns] and the corresponding standard error values for the GFP donors *Sly*LNP1 and *Sly*LNP2. The data show LNP, RHD3 and RTN1 interaction proteins compared with *Sly*LNP1 or *Sly*LNP2, respectively, without interaction partners (−). The ER-localised transcription factor *At*maMYB-mRFP (AT5G45420) was included as a negative control. Statistical significance was assessed using the Kruskal–Wallis test (*** *p* < 0.001). Lifetimes significantly lower than those of *Sly*LNP1 or *Sly*LNP2 alone indicate protein–protein interactions. Biological replicates *n* = 5 with at least 12 technical replicates per combination.

In Arabidopsis, yeast and mammalian LNP proteins, interactions with other ER shapers have been shown and are suggested to be an important aspect of ER structure maintenance and homeostasis [25,28,31,33]. The formation of protein–protein interactions of tomato LNP proteins with each other and other ER shapers (*Sly*RHD3 and *Sly*RTN1) was tested using Förster resonance energy transfer by fluorescence lifetime imaging microscopy (FRET-FLIM) analysis *in planta* (Figure 5). FRET-FLIM measures the reduction in the excited-state lifetime of GFP (donor) fluorescence in the presence of an acceptor fluorophore (mRFP). A reduction in GFP lifetime is an indication that the two proteins are within a distance of 1–10 nm, thus indicating a direct physical interaction between the two protein fusions [12,13,45]. It was previously reported that a reduction of as little as 200 ps in the excited-state lifetime of the GFP-labelled protein represents quenching through a protein–protein interaction [12,13,45]. Donor and acceptor constructs were co-expressed in *N. tabacum* leaf epidermal cells, and FRET-FLIM analysis was performed after 72 h to assess protein–protein interactions. For both *Sly*LNP1-eGFP and *Sly*LNP2-eGFP, a significant reduction in the donor lifetime fluorescence in the presence of all tested acceptor fluorophores (*Sly*LNP1-mRFP, *Sly*LNP2-mRFP, *Sly*RHD3-mRFP, *Sly*RTN1-mRFP) was observed, in comparison to expression of the LNP donor alone (Figure 5). These data suggest that both *Sly*LNP proteins showed significant interactions not only with themselves but also with each other, suggesting that homo- and heteromeric LNP complexes may exist within the tomato ER membrane. In addition, both *Sly*LNP1 and *Sly*LNP2 interacted with the ER fusogen *Sly*RHD3 and the curvature-stabilising protein *Sly*RTN1. These data showed reduced donor fluorescence lifetimes when *Sly*LNP1 and *Sly*LNP2 were co-expressed with themselves, each other, *Sly*RHD3 or *Sly*RTN1, consistent with close molecular proximity between the corresponding protein fusions. The results suggest that tomato LNP proteins may associate with one another and with other ER-shaping proteins. However, FRET-FLIM alone cannot establish direct binding or demonstrate the formation of a functional protein network. The observed proximity patterns are consistent with a potential role for these proteins in a shared ER-shaping system.

These interaction profiles closely resemble those previously reported for Arabidopsis LNP proteins, where LNPs physically associate with both RHD3 and RTN1 to regulate ER morphology [28,29,31]. Together, these results suggest that the molecular machinery governing ER architecture is highly conserved between Arabidopsis and tomato. Together, these findings demonstrate that tomato LNP proteins form part of the conserved ER-shaping protein interaction network, supporting conservation of the molecular machinery controlling ER architecture.

### 2.6. Arabidopsis but Not Tomato LNP Proteins Enhance Resistance to Phytophthora Infestans

**Figure 6:**
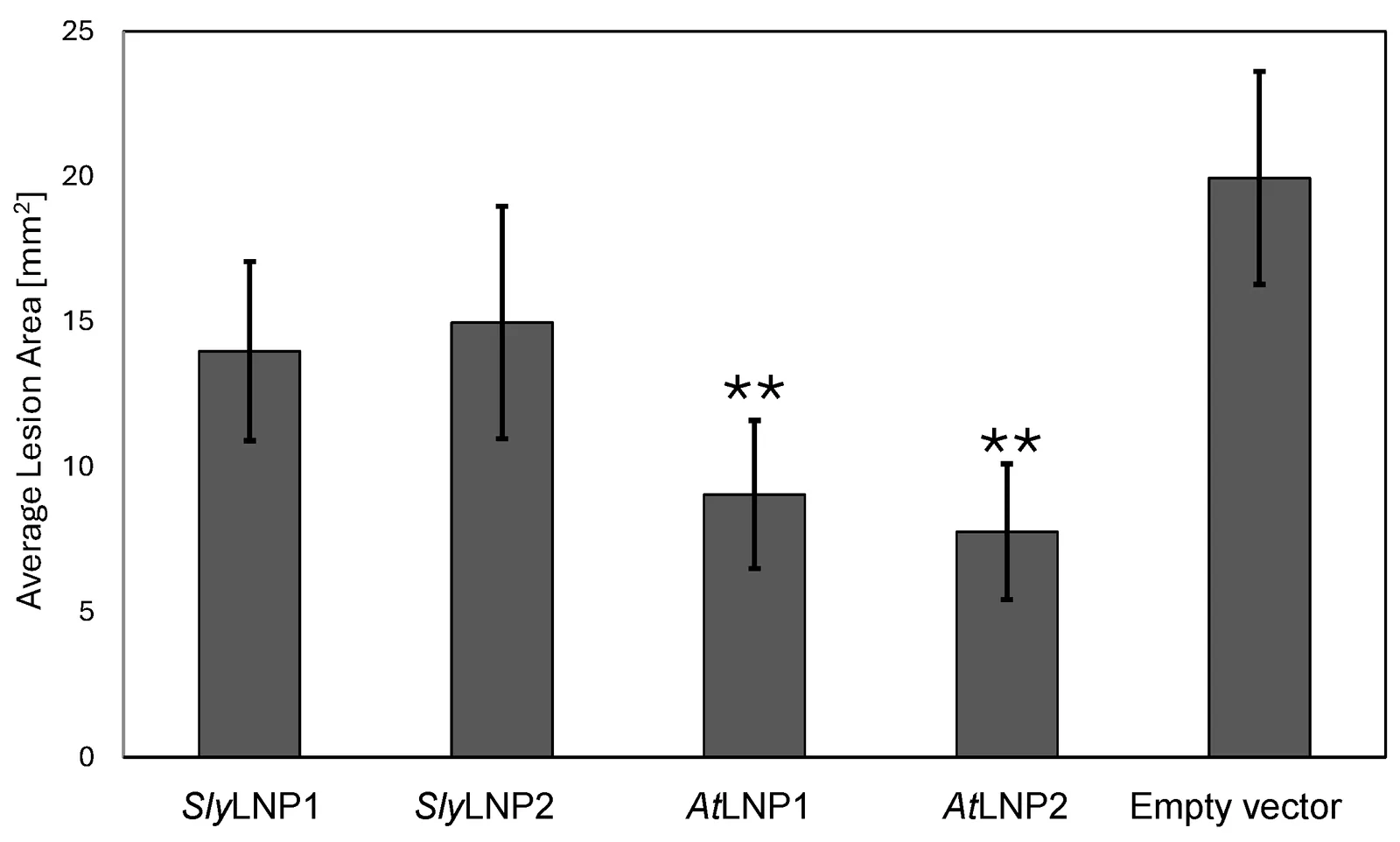
Arabidopsis but not tomato LNP proteins reduce infection by *P. infestans.* Quantitative analysis of *P. infestans* lesion area following transient expression of *Sly*LNP1, *Sly*LNP2, *At*LNP1 or *At*LNP2 in *Nicotiana benthamiana* leaf epidermal cells is presented. Lesion diameters were quantified 7 days after inoculation. *At*LNP1 and *At*LNP2 significantly reduced lesion formation, whereas *Sly*LNP1- and *Sly*LNP2-expressing plants produced lesion sizes comparable to the empty vector control. Data represent the mean ± SE from *n* = 17 biological replicates with 51 technical replicates each. Statistical significance was determined using *p*-values from a two-tailed *t*-test assuming unequal variances (Welch’s *t*-test), and asterisks indicate significant differences (** *p* < 0.01).

Since ER organisation plays important roles in plant immunity and secretory responses during pathogen attack, we investigated whether overexpression of tomato LNP proteins influences susceptibility to the oomycete pathogen *Phytophthora infestans*. Tomato and Arabidopsis LNP proteins were expressed in *N. benthamiana* in a transient manner. Protein expression levels were quantified [29] and showed no overall statistically significant differences. Transient expression of Arabidopsis *At*LNP1 and *At*LNP2 in *N. benthamiana* significantly reduced lesion development following infection (Figure 6). In contrast, expression of *Sly*LNP1 or *Sly*LNP2 did not significantly alter lesion size compared with the control, despite producing pronounced changes in ER morphology and secretory trafficking. These findings indicate that while the ER-shaping function of LNP proteins is conserved, their contribution to pathogen infection is not necessarily transferable between plant species and that conservation of ER-shaping activity does not necessarily result in conservation of downstream physiological functions.

## 3. Discussion

### 3.1. Tomato LNP Proteins Are Conserved Regulators of Plant ER Architecture

LNP proteins have been characterised in fungi, mammals and Arabidopsis [25,28,32], but their functions in crop species have received less attention. The localisation and ER phenotypes observed here are broadly consistent with those reported for *At*LNP proteins [28,29]: *Sly*LNP1 was enriched in ER cisternae, whereas *Sly*LNP2 labelled both cisternae and tubules, and overexpression of either protein promoted cisternal expansion. These similarities support a conserved role for plant LNP proteins in regulating ER organisation. The distinct localisation patterns of the two tomato paralogues may nevertheless reflect differences in how they contribute to ER architecture. Because the proteins were analysed by transient expression in a heterologous host, their behaviour does not establish their endogenous functions in tomato; this will require validation in the native species.

### 3.2. Tomato LNP Proteins Promote ER Cisternal Organisation Rather than Simply Increasing Membrane Abundance

The increase in cisternal area without broad changes in other measured ER network descriptors suggests that LNP overexpression changes ER organisation rather than causing indiscriminate ER proliferation. The redistribution of the luminal marker GFP-HDEL towards cisternal edges in *Sly*LNP1-expressing cells, but not in *Sly*LNP2-expressing cells, further suggests that similarly enlarged cisternae may differ in their internal organisation. This interpretation is consistent with ultrastructural analyses of *At*LNP-induced cisternae, which revealed interconnected tubular matrices associated with flattened membrane domains rather than uniform sheets [29]. The equivalent ultrastructure has not been demonstrated in tomato, so this remains a possible explanation for the fluorescence pattern rather than a direct structural conclusion. Such organisation could influence the distribution of luminal or membrane proteins, or the positioning of ER exit sites, but these possibilities require direct testing.

### 3.3. Conserved Interactions with RHD3 and RTNs Support a Common ER-Shaping Machinery

ER morphology is maintained through coordinated activities of membrane-fusion proteins, membrane-curvature proteins and junctional regulators [25,33]. FRET-FLIM analysis demonstrated that both tomato LNP proteins interact with themselves, each other, *Sly*RHD3 and *Sly*RTN1, indicating that they participate in the conserved ER-shaping protein network. These observations closely parallel previous interaction studies in Arabidopsis, where *At*LNP proteins physically associate with *At*RHD3 and *At*RTN1 [28,31]. Rather than functioning independently, plant LNP proteins therefore appear to act within a dynamic protein complex that balances membrane fusion and membrane curvature. RHD3-mediated membrane fusion likely generates new cisternal domains, whereas RTNs stabilise highly curved tubules. LNP proteins may coordinate these opposing activities by promoting stabilisation and maturation of newly formed cisternal regions. Such a model is consistent with observations from yeast and mammalian systems, although the precise localisation and functional output of plant LNP proteins clearly differ from their non-plant counterparts [28,31].

### 3.4. ER Architecture Is Closely Linked to Secretory Pathway Function

One of the most important findings of this study is that alterations in ER morphology correlate with reduced secretory pathway efficiency. Both *Sly*LNP proteins significantly impaired ER-to-Golgi transport despite producing only moderate changes in overall ER geometry. These observations reinforce the concept that ER architecture is intrinsically linked to ER function rather than representing a passive structural scaffold [2]. Plant ER exit sites are closely associated with mobile Golgi bodies, forming functional secretory units that facilitate rapid protein export [5,7]. Expansion of cisternal domains could alter the spatial organisation of ER exit sites or influence cargo concentration prior to export. Similar reductions in ER–Golgi transport have recently been reported following Arabidopsis LNP overexpression, where ultrastructural remodelling of cisternae was proposed to alter secretory efficiency [29]. Together, these studies suggest that LNP-mediated control of ER morphology directly regulates secretory capacity and that this relationship is conserved across diverse plant species.

### 3.5. Conserved ER Remodelling Does Not Necessarily Translate into Conserved Infection Outcomes

Despite their conserved effects on ER architecture and protein interactions, tomato LNP proteins did not significantly affect lesion development following *Phytophthora infestans* infection, whereas expression of Arabidopsis LNP proteins reduced lesion formation. These findings suggest that conserved molecular functions do not necessarily translate into equivalent effects on pathogen responses. The ER is central to plant immunity because it supports synthesis, folding and secretion of pattern-recognition receptors, antimicrobial proteins and defence-related cell wall components [35,36]. It might therefore be expected that proteins influencing ER organisation would also affect infection by pathogens. However, ER remodelling alone may be insufficient to enhance resistance, and its effects may depend on species-specific interaction partners, regulatory pathways and defence signalling networks.

One possible explanation for the contrasting pathogen phenotypes is that the effects of LNP-mediated ER remodelling depend not only on changes in ER architecture but also on how these changes influence the trafficking of defence-related proteins. Both tomato LNP proteins reduced ER-to-Golgi transport, which could potentially affect the delivery of immune receptors, antimicrobial proteins or defence-associated cell wall components. However, the consequences for resistance will depend on which cargoes are affected and how their altered trafficking influences the defence response. Furthermore, although *Sly*LNP1 preferentially localised to cisternae and altered the distribution of the ER luminal marker, whereas *Sly*LNP2 was distributed more broadly throughout the ER network, both proteins produced similar outcomes in the pathogen assay. This suggests that the observed differences in ER organisation do not necessarily translate into differences in resistance. The contrasting outcomes observed with Arabidopsis and tomato LNP proteins could also reflect differences in protein accumulation or interactions with host-specific regulatory pathways. These possibilities remain speculative, as protein abundance and the effects of LNP expression on immune signalling and defence-associated cargo trafficking were not directly assessed. Several limitations of the present assay should be considered. LNP proteins were transiently expressed in *Nicotiana benthamiana*, a heterologous host, meaning that the observed responses may not fully reflect their functions in tomato or Arabidopsis under native conditions. In addition, disease development was assessed by lesion area at a single time point, seven days after inoculation, which does not capture infection dynamics or distinguish the effects on pathogen proliferation from the effects on host tissue damage. The lack of a significant effect of tomato LNP proteins on lesion area therefore does not exclude more subtle or context-dependent roles in immunity. Future studies combining time-course analyses, direct measurements of pathogen biomass and investigations in native host systems with quantitative proteomics and interactome analysis during infection should help clarify how ER organisation influences plant defence and identify the pathways that determine whether ER remodelling affects pathogen resistance.

### 3.6. Implications for Crop Improvement

The demonstration that tomato LNP proteins regulate ER organisation similarly to Arabidopsis extends current understanding of plant ER biology from a model species into an agriculturally important crop. Since the ER underpins secretion of cell wall components, membrane proteins and defence molecules, engineering ER architecture represents an attractive strategy for improving plant productivity and resilience. However, the contrasting pathogen phenotypes observed here suggest that manipulation of ER morphology alone is unlikely to provide broad-spectrum disease control. Instead, future engineering approaches will probably require coordinated modulation of ER-shaping proteins together with downstream components of the secretory and immune pathways. Understanding how ER structure influences membrane trafficking therefore represents an important step towards rational engineering of plant secretory capacity for agriculture and biotechnology.

### 3.7. Conclusions

This study characterises the tomato Lunapark proteins *Sly*LNP1 and *Sly*LNP2 and shows that they share key features with Arabidopsis LNP proteins in their subcellular localisation and effects on ER architecture. Overexpression of both tomato proteins promoted ER cisternal expansion and reduced ER-to-Golgi trafficking. FRET-FLIM revealed close molecular proximity between the LNP proteins and the ER-shaping proteins *Sly*RHD3 and *Sly*RTN1, consistent with potential associations within the ER-shaping machinery, although direct binding and functional complex formation remain to be established. In the pathogen assay, expression of Arabidopsis LNP proteins reduced *Phytophthora infestans* lesion development, whereas tomato LNP proteins did not produce a significant reduction under the conditions tested. These findings highlight that conserved effects on ER organisation do not necessarily translate into equivalent physiological outcomes.

The findings should be interpreted in light of the use of transient expression in heterologous plant systems, the limitations of FRET-FLIM as evidence of protein association, and the assessment of pathogen responses using lesion area at a single time point. Future work should validate the proposed protein associations using independent approaches, establish the effects of LNP proteins in their native host species, and use time-course infection assays and direct measurements of pathogen biomass to clarify their contributions to plant defence. Further investigation of the molecular pathways linking ER architecture, secretory trafficking and immunity will help determine how conserved ER-remodelling functions influence plant physiology and whether they can be exploited to improve crop performance and resilience.

## 4. Materials and Methods

### 4.1. Sequence Alignment and Structural Analysis of SlyLNP Proteins

Genes encoding for *Sly*LNP1 and *Sly*LNP2 were identified in the National Centre for Biotechnology Information (NCBI) database. Protein sequences for *Sly*LNP1, *Sly*LNP2, *At*LNP1 and *At*LNP2 were identified using BLASTP (version 2.17.0) searches [46] and retrieved for comparative analysis. Multiple sequence alignment was performed using Clustal Omega version 1.2.4 [47]. Predicted structural features of *Sly*LNP1 and *Sly*LNP2 were compared with those of *At*LNP1 and *At*LNP2. Transmembrane domains (TMDs) were predicted using TMHMM 2.0 [48]. Coiled-coil domains (CCDs) were predicted using the NPSA Coiled-Coil Prediction tool, based on the method of Lupas et al. [49]; regions with a predicted probability >0.5 using a 28-residue scanning window were included. The C-terminal zinc-finger region was identified from the conservation of the characteristic four cysteine residues in the multiple sequence alignment. Amino acid conservation was calculated from the Clustal Omega multiple sequence alignment [47] and visualised using Jalview version 2.11.5.3 [50].

### 4.2. Construct Generation and Gene Identities

*Sly*LNP1, *Sly*LNP2 and *Sly*RHD3 were commercially synthesised and cloned into Gateway-compatible pENTRY vectors using Twist Bioscience (South San Francisco, CA, USA). Genes were then cloned into pB7FWG0 (C-terminal mCherry fusions), pB7RWG2 (C-terminal mRFP fusions) and pB7FWG2 (C-terminal eGFP fusions) destination vectors [51] using Gateway technology (Invitrogen; Thermo Fisher Scientific, Waltham, MA, USA). The sequences of these genes and their loci names (available on the Sol Genomics Network [52]) are detailed in Supplementary Table S3. Other vectors in this study have been published previously, including RFP-HDEL, GFP-HDEL [43], ST-GFP [44] and SP-mCherry [53].

### 4.3. Transient Gene Expression in Tobacco

For Agrobacterium-mediated transient expression, 5–6-week-old tobacco (*Nicotiana tabacum* cv. Petit Havana SR1) plants grown in the glasshouse with a 12 h light:dark cycle were used. Transient expression was performed according to [35]. In brief, each construct was introduced into *Agrobacterium tumefaciens* strain GV3101 by heat shock. Transformants were inoculated into 3 mL of LB medium (10 g tryptone, 5 g yeast extract, 10 g NaCl in 1 L deionised H_2_O) with 50 μg mL^−1^ spectinomycin and 25 μg mL^−1^ rifampicin. After overnight shaking at 28 °C, 2 mL of the bacterial culture was pelleted by centrifugation at 2200× *g* for 5 min at room temperature. The pellet was washed twice with 1000 μL of infiltration buffer (50 mM MES, 2 mM Na_3_PO_4_ 12H_2_O, 0.1 mM acetosyringone and 5 mg mL^−1^ glucose) and then resuspended in 1 mL of infiltration buffer. The bacterial suspension was diluted to a final optical density at 600 nm (OD_600_) of 0.05–0.2. Tobacco leaves were then gently infiltrated on the abaxial side using a syringe without a needle. Infiltrated plants were then returned to glasshouse conditions for 72 h before imaging.

### 4.4. Confocal Microscopy

For images collected using confocal microscopy, a small leaf piece from the infiltrated area (approximately 5 mm^2^) was excised with a scalpel and placed onto a glass slide with the abaxial side facing up. Images were acquired using a Zeiss LSM880 confocal microscope with an Airyscan detector (Carl Zeiss Microscopy GmbH, Oberkochen, Germany), either a Zeiss Plan-Apochromat 100×/1.46 NA oil-immersion objective or a Plan-Apochromat 63×/1.46 NA oil-immersion objective. GFP was excited at 488 nm, and emission was collected at ~523 nm; RFP and mCherry were excited at 561 nm, with emission detected at ~579 nm. All images were acquired in line-switch scanning mode with 2–4× frame averaging to enhance signal quality.

### 4.5. ER Structure Analysis

The AnalyzER V 1.1 software package [41,42] was used to quantify changes in ER morphology induced by *Sly*LNP1 and *Sly*LNP2 overexpression. GFP-HDEL was co-expressed alongside *Sly*LNP1 and *Sly*LNP2 to label the ER, and for the control group, RFP-HDEL was co-expressed with GFP-HDEL. ER structure was analysed as described in [42]. Briefly, the tubule minimum full width at half maximum (FWHM_min_) was set to 3 pixels; the maximum full width at half maximum (FWHM_max_) was set to 10 pixels; the target tubule width was set to 5. The background was subtracted, a network template image was produced, and a phase-congruency-based enhanced image was made. The phase-congruency-based enhanced image allowed for the accurate identification of tubules in an intensity-dependent manner because segmentation based on intensity can produce errors and discontinuous tubule labelling [54]. Following this, a single-pixel skeleton was made from the enhanced image, and the cisternal boundaries were labelled. Cisternal regions are identified by an opening function, followed by refinement onto the underlying image intensity. As described by [37], cisternal areas are regions larger than 0.3 µm^2^ and are characterised as tubule junctions and static points, most likely correlating with ER–plasma membrane contact sites. Polygonal regions are defined as regions of cytoplasm that are fully enclosed by the ER. A total of 9 ER structure metrics, covering the structure and geometry of different ER subdomains, were calculated for comparison of ER structures (Supplementary Table S4).

Statistical analysis was performed using a custom-designed software package designed to accompany AnalyzER [42]. Initial differences between the ER structures induced under overexpression of *Sly*LNP1 and *Sly*LNP2 were identified by a MANOVA. Once differences in the overall ER structures induced by *Sly*LNP1 and *Sly*LNP2 were confirmed, the specific parameters responsible for these differences were confirmed by multiple ANOVA comparisons with a Bonferroni correction applied to each *p*-value to account for multiple comparisons. Sample sizes: control, *n* = 17; *Sly*LNP1, *n* = 24; *Sly*LNP2, *n* = 26; data derived from three independent biological replicates.

### 4.6. Cisternae Structure Analysis

Cisternal boundaries were identified using AnalyzER’s ‘thresh1’ segmentation method, which removes tubular ER elements through greyscale morphological opening using a radius 20% greater than the FWHM_max_ of the tubules, followed by Otsu’s automatic thresholding to generate a binary mask. The resulting segmentation was refined using the active-contour algorithm to align the detected cisternal boundaries with the underlying fluorescence intensity distribution. The cisternal edge was therefore defined by the perimeter of the refined binary segmentation rather than by a manually selected fluorescence intensity threshold. Using AnalyzER’s inbuilt function to assess the intensity at cisternae boundaries [42], fluorescence intensity was measured along radial transects perpendicular to the segmented cisternal boundary, extending both outwards from the edge and inwards towards the cisternal interior. The segmented boundary was used as the reference position for each transect, allowing fluorescence intensity to be compared as a function of distance from the cisternal edge. Only images with a minimum of 5 identifiable cisternae were used for analysis to prevent domination of small cisternae within the analysis. Intensity profiles were calculated over a fixed distance from the segmented cisternal boundary, irrespective of cisternal size, rather than being normalised to the dimensions of individual cisternae. The mean intensity along each cisterna in each image was aggregated to give a singular measure of intensity from the outside of a cisterna to inside a cisterna in the control cells and on overexpression of *Sly*LNP1 and *Sly*LNP2. Data presented are means from *n* = 7 (control), *n* = 9 (*Sly*LNP1), and *n* = 11 (*Sly*LNP2) cells, collected from three independent biological replicates.

### 4.7. ER–Golgi Transport Analysis

Fluorescence images were analysed using Fiji (ImageJ 1.54p) [55]. Regions of interest (ROIs) corresponding to background, ER, and Golgi compartments were manually delineated. A background ROI was selected in a region devoid of fluorescence to correct for background signal. For each cell, three Golgi ROIs and three ER ROIs were drawn in the green channel to quantify mean intensity. In cases where the ER was not clearly visible in the green channel, the corresponding regions were identified using the red channel. If fewer than three Golgi bodies were visible in a cell, two (or fewer, when necessary) Golgi ROIs were selected in spatial proximity to the ER. Mean gray-value intensities for each ROI were measured using Fiji’s Analyze → Set Measurements → “Mean gray value” function. Background intensity was subtracted from both ER and Golgi measurements. Finally, the ratio between the mean intensity of the ER and Golgi was computed for each cell. Data were collected from *n* = 11 (control), *n* = 12 (*Sly*LNP1), and *n* = 14 (*Sly*LNP2) cells, collected from three independent biological replicates.

### 4.8. FRET-FLIM

In brief, 5 mm^2^ of transformed leaf section was placed on a FRET-FLIM microscope built around a Nikon Ti2 inverted microscope (Nikon Corporation, Tokyo, Japan), with the abaxial side facing the objective. Excitation was produced with an NKT super continuum laser (SuperK EXTREME NKT-SC; NKT Photonics, Birkerød, Denmark) together with a SuperK SELECT multi-line tunable filter (Photonic Solutions, Edinburgh, Scotland, UK), emitting 70 ps pulses at 80 MHz, and focused with a water-immersion Nikon VC/1.2 NA objective to illuminate specimens on the microscope stage. GFP and RFP/mCherry were excited at 491 nm (emission 520/35 nm) and 561 nm (emission 630/50 nm), respectively. The detector (hybrid PMT, GaAsP, HPM-100-40, Becker & Hickl GmbH, Berlin, Germany) was linked to a time-correlated single-photon counting PC module (SPC-QC-104, Becker & Hickl, Berlin, Germany) to generate the raw FRET-FLIM data. A 633 nm interference filter (633IU, Comar Optics Ltd., Haverhill, UK) was used to minimise the autofluorescence emission of chlorophyll, which would otherwise obscure the fluorescence emission of RFP, mCherry and GFP. The FLIM parameters were: a large pinhole, 100 µm field zoom, 5 µsec pixel dwell time and a 256 × 256 pixel area. Image data were analysed with binning set to no more than 3 in the analysis software. The data were analysed using SPCImage 8.9 Software (Becker & Hickl GmbH, Berlin, Germany). Lifetime exponential decay curves were extracted for every pixel within a region of interest around the ER and Golgi to calculate the average excited-state lifetime values per cell. Only cells with a χ2 between 0.9 and 1.4 were used; at least 12 nuclei from at least 5 biological samples were analysed per protein–protein combination, and the average of the ranges was taken.

### 4.9. Agrobacterium Tumefaciens Transient Assay (ATTA)

Four-week-old *N. benthamiana* plants (ongoing lab stocks) were grown under greenhouse conditions, and ATTAs were conducted essentially as described by [56,57]. Briefly, the second, third and fourth true leaves were used from the *N. benthamiana* plants, and *Agrobacterium tumefaciens* carrying plasmids containing *SlyLNP1*, *SlyLNP2*, *AtLNP1*, *AtLNP2* and the empty vector (pB7WGF2) were infiltrated at OD = 0.1, as described by [35].

**Figure 7:**
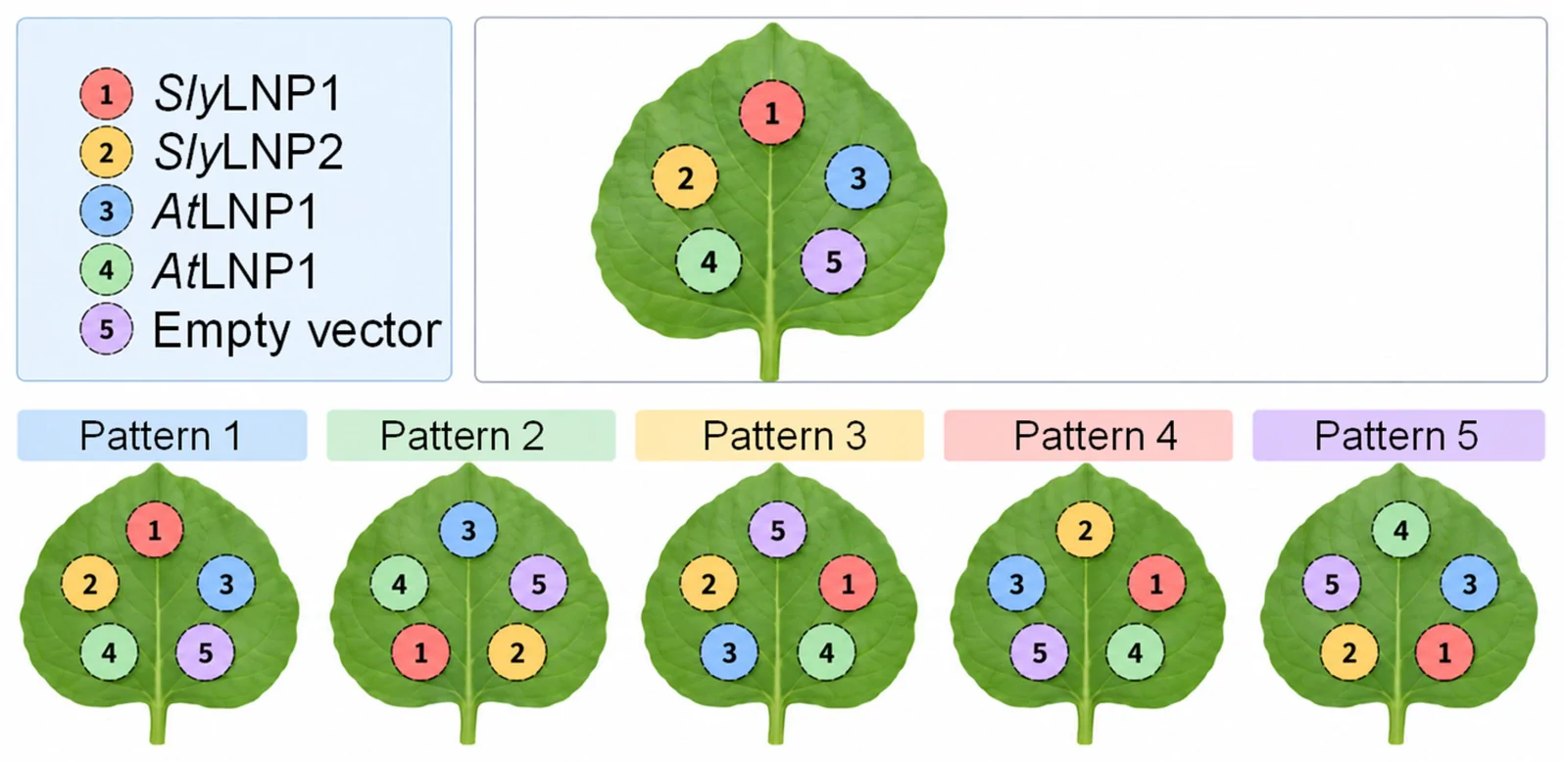
Experimental design and infiltration patterns used for the *Phytophthora infestans* infection assay. Schematic representation of the five infiltration patterns used to compare transient expression of *Sly*LNP1, *Sly*LNP2, *At*LNP1, *At*LNP2 and the empty vector control in *N. benthamiana* leaves. The spatial arrangement systematically varied across five patterns to minimise potential positional effects associated with infiltration site.

The pathogen assay was performed using 17 plants, with five infiltration sites on each leaf corresponding to the five experimental treatments *(Sly*LNP1, *Sly*LNP2, *At*LNP1, *At*LNP2 and the empty vector control; Figure 7). The *Phytophthora infestans* infection assay was based on established transient-expression assays in *Nicotiana benthamiana* [58,59], with modifications to accommodate the simultaneous comparison of five treatments on each leaf.

Three leaves per plant (the second, third and fourth true leaves) were used, with all five experimental treatments infiltrated at separate sites on each leaf. This resulted in 51 leaf-level observations per treatment across the 17 plants. The five treatments were present on each leaf, allowing within-leaf comparisons under shared plant-level conditions. The spatial arrangement of treatments was systematically varied using five different infiltration patterns to minimise potential positional effects. Treatment allocation was structured rather than randomised, and blinding was not performed. The three leaves from each plant were treated as technical replicates, with the plant representing the biological replicate.

The plants were kept in a growth cabinet on a 16 h light cycle at 22 °C for 24 h, and zoospores from *P. infestans* wild-type strain 3928A (James Hutton Institute’s Phytophthora culture collection), cultured as described in [60], were collected for plant infection. At each infiltrated site, 10 µL of zoospores (200,000 zoospores mL^−1^) were applied. Once inoculated, the leaves were placed in a dark cabinet (21 °C, 55% humidity) for the initial 18 h post-inoculation. Dark cabinet conditions were removed, and the inoculated *N. benthamiana* leaves were kept in a growth cabinet on a 16 h light cycle at 22 °C. Lesion area was measured at 7 days post-infiltration. Bar plots present lesion area relative to that for the control. A two-tailed *t*-test assuming unequal variances (Welch’s *t*-test) was performed to identify statistically significant differences. Data are presented from *n* = 17 biological replicates with 51 technical replicates per construct. This was achieved by infiltrating the same leaf five times to transiently express *At*LNP1, *At*LNP2, *Sly*LNP1, *Sly*LNP2 and the empty vector control. The five infiltrations were arranged in five different permutations across the 51 technical repeats to ensure that bias due to infiltration location, inoculation site and/or leaf was removed.

## Data Availability

Dataset available on request from the authors.

## Supplementary Materials

The following supporting information can be downloaded at: https://www.mdpi.com/article/10.3390/plants15213447/s1, Figure S1: Full sequence amino acid alignment of tomato LNP proteins with Arabidopsis, yeast and human LNP proteins; Table S1: ANOVA results from each ER structural characteristic investigated; Table S2: ANOVA *p*-values are adjusted with the Bonferroni correction to allow for multiple comparisons; Table S3: Names and sequences of proteins used in this study; Table S4: Definition of the 9 metrics used to assess ER structure.

## Abbreviations

The following abbreviations are used in this manuscript:

ER	endoplasmic reticulum
LNP	Lunapark
ERES	ER exit sites
RTN	reticulon
RHD3	ROOT HAIR DEFECTIVE 3
TMD	Transmembrane domain
ST	Sialyl Transferase
CCD	coiled-coil domains
FWHM_max_	maximum full width at half maximum
FWHM_min_	minimum full width at half maximum
ROI	Region of interest
ATTA	*Agrobacterium tumefaciens* Transient Assay
