# Supplementary material for: Conserved Architecture, Divergent Function: Tomato Lunapark Proteins Remodel the Endoplasmic Reticulum Without Enhancing Pathogen Resistance
Source: Plants (Basel). 2026 Nov 5;15(21):3447. doi: 10.3390/plants15213447 (PMC13765813; doi:10.3390/plants15213447)
Supplement: Supplementary file 1 [file plants-15-03447-s001.zip › plants-4620165-supplementary.pdf]

Supplementary Data:

Conserved architecture, divergent function: tomato Lunapark proteins remodel the endoplasmic reticulum without enhancing pathogen resistance

```
scLNP1p       -----------------------------------------------------------M  1
HsLNP         MGRSWPGLSLCQWRLLPALEAGWA-VAAETGGGGRPGRAAPASCSCEFLWTIDDIYHCAQ  59
SlyLNP1       ------------------MAAEPTKEHIDV------AETEPVASSTDNEEKTKKKKKKGF  36
SlyLNP2       ------------------MATESEKDLGDT------AGAAP-VTSTDNEAKTKKKKKKGL  35
AtLNP1        ---------------MAVGEKEHEGTVVESGG-EKNDSAVVLS----ASGEKKTTKRKGL  40
AtLNP2        ------------------MAQEQEGAVVEKGE-PNDSSAAAVAATATATADSVKKKQNGF  41

scLNP1p       FSAL-GKWVRGSRN-DKDFVTKYTADLSQITSQIHQLDVALKKSQSILSQWQ---SNLTF  56
HsLNP         FLQIKDGW-IIFSMEGKTK-PSTVEVLESIDKEIQALEEFREKNQRLQKLWV--GRLILY  115
SlyLNP1       FS---MIWNSLFRSKKDDF----EKRLQHISKEEAAVIA---RINKRSQNWRRMTRHLIV  86
SlyLNP2       LS---RMWNSLFGSHKDDF----EKRLQHISKEEAAVIA---RTVKRSHSWKRMSRHLII  85
AtLNP1        FS---RLWNAIFRVRGDDF----EKRLKNISKEEATVRN---RMKRRSITRRNFIRNLIA  90
AtLNP2        FS---RLWNGIFRVRGDDF----EKRLQYISREEATVLS---RMKRRSISWRKLTRNLIV  91
              :      *        .         *. *  :   :     :              :

scLNP1p       YGIALTVLALSYTYW----EYHGYRPYLVVTALLCIGSLILFKWALTKLYAFYNNNRL   110
HsLNP         SSVLYLFTCLIVYLWYLPDEFTARLA--MTLPFFAFPLIIWSIRTVIIFFFSKRTERNN  172
SlyLNP1       LSVIFEVIAVGYAIMTTRSLELNWKMRALRVLPMFLLPGLSFITYSAIGSFTRMCERKDQ  146
SlyLNP2       LSVLFEVIAMAYAIMTTRSLELNWKMRALRVLPMFLLPGLSFFTYSTLRSFTRMKEQRDQ  145
AtLNP1        FSVFFEVIAVSYAIMTTRDEDLDWKLRSFRILPMFLLPAVAFLLYSSLVGFWRMCDRRDQ  150
AtLNP2        SSVLFEIIAVGYAILTTRTEDLDWRMRSFRILPMFILPAVSALAYSSIVSFSKMFDRRDQ  151
               .:   . .:             :       :  :            :  :     ..

scLNP1p       RKLAKLRAIHQKKLEKLKEETHYNATSSII------------------------------  140
HsLNP         EALDDLKSQRKKILEEVMEKETYKTAKLIL------------------------------  202
SlyLNP1       KTLEKLRAERQAKIEELKEKTNYYITQQLI------------------------------  176
SlyLNP2       KTLERLRAERQAKIDELKEKTNFYITQQLI------------------------------  175
AtLNP1        HTLEKLQAEMLGKINELKERTNYYITQQLI------------------------------  180
AtLNP2        KTLEKLRAERLAKINELKERTNYYTTQQLIQAWHSHATKERSMFADLKRLTTACRAGANR  211
              . *  *::     :::: *.   :  :. ::

scLNP1p       ----QRFSSGEDQNDDAMVLL-DDELNAK------------------YQELNN-------  170
HsLNP         ----ERFDPDSKKAKECEPPSAGAAVTARPGQEIRQRTAAQRNLSPTPASPNQGPPPQVP  258
SlyLNP1       ----QRYDPDPAAKAAAATVL-ASKLGTDTGLKVYVGD--DTKHNVPTGKSNG---VEVV  226
SlyLNP2       ----QRYDPDPASKAAAATVL-ASKLGADTGLKVYLGD--ESKFNVPAGTSND---VAIQ  225
AtLNP1        ----QRYDPDPAAKAAAATVL-ASKLGAESGLKVFVGD--ESQLEPTAGKNNA------K  227
AtLNP2        IVLMQRYDPDPAAKAAAATVL-ASKLGADSGLKVYLGD--ESQLDPSSGKSND---MEVN  265
                  :*:. .      .        : :                       *

scLNP1p       ----LK--------------TELE--------KFKKESH-------V------------K  185
HsLNP         VSPGPPKDSSAPGGP--PERTVTP-----ALSSNVLPRHLGSPATSVPGMGLHPPGPPLA  311
SlyLNP1       QSNGLRNRKQARSSS--PESAVIDHPGAEMLQQAQLE---GSDMMQHQQTVVEHYN---P  278
SlyLNP2       QSAGLRNRKQARSGS--AGSAVLDHREGEMLHDLQLE---GSIMNQHQQMIVEHYN---P  277
AtLNP1        HSGGLRNRKQTNTRGNSAETTPIHHSDNESNHSGTSE-RITGT-EQNQQMVFEHYN---P  282
AtLNP2        QSRGLRNRRQPNTRPHGSGSTSTHHSDDESHHSGTSE-RFPGTTEQNQQMLVEHYS---P  321
                                  :           .

scLNP1p       GLKKEDSDAWFDKIISVLAGGNELDSTSSLSPFKKIICPQCHWKSNCYRLAS-KPIIFIC  244
HsLNP         RPILPRERGALDRIVEYLVGDGPQNR-------YALICQQCFSHNGMALKEEFEYIAFRC  364
SlyLNP1       TGSSTQDGGWIARIAALLVGEDPTQS-------YALICGNCHMHNGLARKEDFPYITYYC  331
SlyLNP2       TSVSTQDGGWLARIAALLVGEDPTQS-------YALICSNCHMHNGLARKEELPYITYYC  330
AtLNP1        QEYAAHDGSWISRIAALLVGEDPSQS-------YALICGNCRMHNGLARKEDFPYITYYC  335
AtLNP2        QGYAAHDGSWISRIAALLVGEDPTQS-------YALICGNCRMHNGLARKEDFAYITYYC  374
                    . . : :*    *.*  .  :          :** :*  :..     .   * : *

scLNP1p       PHCNHKIDEVKEREDAIEAKQPAQPSQSEKEKTK--------------------------  278
HsLNP         AYCFFLNPARKTRPQAPR-----LPEFS----FEKRQVVEGSSS-----------VGPLP  404
SlyLNP1       PHCHALNRPKQLDDRVSGTSTPNLGST---TSLADVDSVKQVSGSTPDKIPASASGSPVA  388
SlyLNP2       PHCNALNGPKPLADGTSS--LLNMAST---TIVADADLVKQDSGSIAERDSASSS--P--  381
AtLNP1        PHCRALNKPKHSEEHSLIAPADTLPKVSLKP--MESEVIN-SSSSTSERGN-----SPIP  387
```

```
AtLNP2        PHCNALNKPKHSEENVLLPAISASPITDSLPLIETSEVVN-SSSSSSERGS-----SPTP  428
                :*

scLNP1p       ------------------------------------------------------------  278
HsLNP         SGSVLSSDNQFNEESLEHDVLDDNTEQTDDKIPATEQTNQVIEKASDSEEPEEKQETENE  464
SlyLNP1       APVETEGDNI-------------------------------VSSAS---NS---------  405
SlyLNP2       AGETTEGNSI-------------------------------VSESS---SPAVVATATTE  407
AtLNP1        -------------------------------------------LLH---TPEIVEEVPE-  400
AtLNP2        -------------------------------------------EIK---EEAAITETGT-  441


scLNP1p       ------------------------------- 278
HsLNP         EASVIET-NSTVPGADSIPDPELSGESLTAE 494
SlyLNP1       ------------------------------- 405
SlyLNP2       DENIVSASSSPAVAALETPEGDHIGSSISSS 438
AtLNP1        ------------TAENETPN----------- 408
AtLNP2        ------------------PS----------- 443
```

Supplementary Figure S1: Full sequence amino acid alignment of tomato LNP proteins with Arabidopsis, yeast and human LNP proteins

Alignment of scLNP1p*, Hs*LNP*, Sly*LNP1 and *Sly*LNP2 and *At*LNP1 and*At*LNP2 produced by Clustal Omega. Transmembrane domains are indicated in blue, zinc finger motives in pink and the LNP motif in yellow.

Supplementary Table S1: ANOVA results from each ER structural characteristic investigated

Comparison of multiple investigated ER structure metrics with the transformation applied to each set of data for normality. P-values of ANOVAs are also shown with p-values adjusted using a Bonferroni correction for multiple comparisons.

**ER subdomain**	**Parameter**	**Applied transformation**	**F statistic**	**p-value (adjusted by a Bonferroni correction)**
Tubules	Length	Logit	0.82	0.45
Cisternae	Area	Logit	7.62	1.08×10^{-3}
	Circularity	Logit	0.38	0.68
	Elongation	Arcsin	1.97	0.15
	Roughness	Arcsin	1.44	0.25
Polygonal region	Area	Logit	5.22	7.93×10^{-3}
	Circularity	Logit	1.42	0.25
	Elongation	Arcsin	1.76	0.18
	Roughness	Logit	1.07	0.35

Supplementary Table S2: ANOVA p-values are adjusted with the Bonferroni correction to allow for multiple comparisons

Statistically analysis of cisternae area and polygonal region area using firstly an ANOVA with a p-value adjusted by the Bonferroni correction for multiple comparisons. Post hoc comparisons are performed using Tukey's test. *** indicates a p-value < 0.001, ** indicates a p-value < 0.01 and * indicates a p-value < 0.05.

ER subdomain	Variable	ANOVA (adjusted p-value)	Post hoc comparison	
			**Control vs *Sly*LNP1 (p-value)**	**Control vs *Sly*LNP2 (p-value)**
Cisternae	Cisternae area	1.08×10^{-3}	5.86×10^{-3}	1.31×10^{-3}
			**	**
Polygonal region	Polygon area	7.93×10^{-3}	9.71×10^{-3}	2.35×10^{-2}
			**	*

Supplementary Table S3: Names and sequences of proteins used in this study

List of unpublished proteins used in this study (*Sly*LNP1, *Sly*LN2, *Sly*RTN1 and *Sly*RHD3) alongside the given sequence and the loci name listed on the Sol Genomics Network.

**Gene name**	**Protein sequence**
*Sly*LNP1<br>Solyc04g064810	MAAEPTKEHIDVAETEPVASSTDNEEKTKKKKKKGFFSMIWNSLFRSKK<br>DDFEKRLQHISKEEAAVIARINKRSQNWRRMTRHLIVLSVIFEVIAVGYAI<br>MTTRSLELNWKMRALRVLPMFLLPGLSFITYSAIGSFTRMCERKDQKTL<br>EKLRAERQAKIEELKEKTNYYITQQLIQRYDPDPAAKAAAATVLASKLGT<br>DTGLKVYVGDDTKHNVPTGKSNGVEVVQSNGLRNRKQARSSSPESAV<br>IDHPGAEMLQQAQLEGSDMMQHQQTVVEHYNPTGSSTQDGGWIARIA<br>ALLVGEDPTQSYALICGNCHMHNGLARKEDFPYITYYCPHCHALNRPK<br>QLDDRVSGTSTPNLGSTTSLADVDSVKQVSGSTPDKIPASASGSPVAA<br>PVETEGDNIVSSASNS
*Sly*LNP2<br>Solyc06g054650	MWNSLFGSHKDDFEKRLQHISKEEAAVIARTVKRSHSWKRMSRHLIILS<br>VLFEVIAMAYAIMTTRSLELNWKMRALRVLPMFLLPGLSFFTYSTLRSFT<br>RMKEQRDQKTLERLRAERQAKIDELKEKTNFYITQQLIQRYDPDPASKA<br>AAATVLASKLGADTGLKVYLGDESKFNVPAGTSNDVAIQQSAGLRNRK<br>QARSGSAGSAVLDHREGEMLHDLQLEGSIMNQHQQMIVEHYNPTSVS<br>TQDGGWLARIAALLVGEDPTQSYALICSNCHMHNGLARKEELPYITYYC<br>PHCNALNGPKPLADGTSSLLNMASTTIVADADLVKQDSGSIAERDSASS<br>SPAGETTEGNSIVSESSSPAVVATATTEDENIVSASSSPAVAALETPEG<br>DHIGSSISSS
*Sly*RTN1<br>Solyc01g095200	MAEHVENSDPKGESLLEKIADKFHGDDSSSSSESDTETKKPAVKSEVE<br>PETETLKNKVWRLFGREKPVHKVFGGGKPADVFLWRNKKISATVIGGA<br>TAIWVLFELLEYHLLTLICHILIITLAVLFLWSNATTFINKKPPHIPEVHLPE<br>GPILQVASALRIEINRALDLLREIASGRELKKFLAVIAGLWILSIVGSWCNF<br>LTLFYISFVLLHTVPVIYEKYEDKVDPLAEKAMHEIKKQYAVFDEKVLSKI<br>PRGPLKDKKKD
*Sly*RHD3<br>Solyc03g121590	MDNKDECCSTHLIDGDGMFNVAGVENFMKEVKLAECGLSYAVVSIMG<br>PQSSGKSTLLNHLFHTNFREMDAYKGRSQTTKGIWMARCAGIEPCTLV<br>MDLEGTDGRERGEDDTAFEKQSALFALAVSDIVLINMWCHDIGREQAA<br>NKPLLKTVFQVKYIAV*SLY*EHSFRMFYNLLFFDQVMMRLFSPRKTTLMF<br>VIRDKTRTPLENLEPVLREDIQKIWDSVPKPQAHKDTPLSEFFNVEVVAL

	SSFEEKEEQFKEQVASLRQRFFHSIAPGGLAGDRRAVVPASGFSFSSQ QIWKVIKENKDLDLPAHKVMVATVRCEEIANEKYVSFTENEEWSQLEEA VNSHSVRGFGRKVSSILDACLSEYDVEATFFDEGVRSSKRKHLEEKLL QLVQPAYQSMLGHIRSDAFERFKEAFEKSLKGGKGFALAARECAESFM SHFDEECSDAIIDQAKWDSSRVKDKLRRDVDAHIAEVRSAKLAEVTTLY ETKLNEALAGPVEALLDGAGDDTWPAIRKLLQRETDTAVSGFAAALSGF EMDEESRDNMVLRLKDYARGVVEAKAKEEAGRVLSRMKDRFSTLFSH DQDSMPRIWTGKEDIRAITKTARSASLKLLSVMAAVRLEDESDSIDKVLI VALVDGKAGASSSKSITSVDPLASSTWDEVPPSKTLITPVQCKSLWRQF KTETEYVVSQAIAAQEASKRNNNWLPPPWAIAAMVILGFNEFMTLLRNP LYLGFIFVAYLLFKALWVQMDISGEFRNGVLPGLLSLSTKFLPTIMNLLK RLAEEGQGVANGQPQANPALSSKSFRGSTNDHGDVSTSGTSEVTSEN GTEYSSSSLHDKAQ

Supplementary Table S4: Definition of the 9 metrics used to assess ER structure

The 9 metrics used to analyse changes in ER structure, the majority of which are identical for cisternae and polygonal regions, just applied to different subdomains.

**Parameter**	**Definition**
Length	The length of an ER tubule (continuous length between junctions/cisternae/free ends)
Area	The area of each cisternae/polygonal region
Circularity	The ratio of the area calculated from the cisternae/polygonal regions radius vs the radius calculated from the perimeter
Elongation	The width:length ratio of each cisternae/polyonal region
Roughness	The $perimeter^2$:area ratio of each cisternae/polygonal region
